# Supplementary material for: Human–Chelonian Bond in Italy: An Exploratory Study of Pet Turtle and Tortoise Ownership
Source: Vet Sci. 2025 Aug 18;12(8):773. doi: 10.3390/vetsci12080773 (PMC12390726; doi:10.3390/vetsci12080773)
Supplement: Supplementary file 1 [file vetsci-12-00773-s001.zip › vetsci-3745158-supplementary.pdf]

Supplementary Materials

S1- Full questionnaires translated in English, the questionnaire was administered in Italian**.**

Part 1 – General questions about the animal

Note: If you have more than one lizard/snake/turtle, choose only one animal to answer about – consider only one species and respond for the animal that has been living with you the longest.

- **What is the scientific or common name of the species?** Testudo hermanni; Testudo Graeca; Testudo marginata Testudo horsfieldii; Trachemys; Pseudemys; Graptemys; Sternotherus; Terrapene carolina

- **How old is your reptile? (estimated or actual):** <1; Between 2 and 5; Between 6 and 10; Between 11 and 20; Between 21 and 30; Between 31 and 40; Between 41 and 50; Between 51 and 60; Between 61 and 70; Between 71 and 80; Between 81 and 90; Between 91 and 100; More than 100; I don't know

- **How long ago did you get/purchase it?**

- **Sex: Male • Female • I don't know •**

- **Is this your first reptile (kept as a pet)? Yes • No •**

- **Where did you get/purchase it?:** Pet store; Given as a gift; Caught in the wild; Other (please specify)

- **Have you had other animals in the past?** Dog; Cat; Rabbit; Parakeet; I did not have other animals in the past; Other (please specify)

Part 2 – Questions about housing/care

Definitions:
Terrarium: An enclosure where the environmental conditions (e.g., humidity, lighting, temperature) necessary for terrestrial and semi-terrestrial species are artificially recreated.
Aquarium: A contained space where environmental conditions necessary for aquatic or semi-aquatic species are artificially recreated.
Cage: An enclosure often made of mesh, bars, or wire fencing.

Environment where the reptile lives: Always or almost always free, not in an enclosure/terrarium/aquarium/cage; Partly free and partly in an enclosed space (enclosure/terrarium/aquarium/cage); Mostly in an enclosed space (enclosure/terrarium/aquarium/cage); Only in an enclosed space (enclosure/terrarium/aquarium/cage)**;** Depends on the season

- **Where is it housed? (you may select more than one answer):** Terrarium*; Aquarium*; Cage*;Free-roaming; Other

- **Please specify the approximate size of the enclosure (terrarium/aquarium/cage): (length) _____ cm x (width) _____ cm x (height) _____ cm**

- **How many animals share the enclosure (terrarium/aquarium/cage)?:** It lives alone; 2 animals live in it; 3 animals live in it; Other

- **What other animals share the enclosure (terrarium/aquarium/cage)? (please specify):** Other reptiles of the same group; Other reptiles of a different group; It lives alone; Other

- **The enclosure (terrarium/aquarium/cage) has:** Transparent walls (glass); Reflective walls (mirror-like); Opaque walls; Mixed walls – transparent, reflective, or opaque; Other

- **Does the reptile have access to sunlight or UVB light? (you may select more than one answer):** Yes, sunlight through a glass window; Yes, direct sunlight ; Yes, UVB lamp outside the enclosure; Yes, UVB lamp inside the enclosure; No; I don't know

- **Do you provide a light-dark cycle?:** Yes; No; I don't know

- **Does the enclosure (terrarium/aquarium/cage) have: (you may select more than one answer):** Heat sources; Heating lamps; UVB lamp; Fluorescent lamp; A lamp, but I don't know what type; None of the above; Other

- **What type of heat source do you provide?:** Infrared lamp; Ceramic heat lamp; Mercury vapor lamp; Heating mats; Heating cables; Heat emitters; Other

- **Please specify the temperature in the enclosure:** Warmest point: _____ Coolest point: _____ Average temperature: _____

- **Are there hiding spots in the reptile's environment?:** Yes; No; I don't know

- **If it is a semi-aquatic turtle, does it have access to dry platforms or areas?:** Yes; No; I don't know

- **What kind of substrate is in the enclosure? (you may select more than one answer):** Water (enough to swim in); Shallow water (enough to bathe in); Sand; Wood shavings; Bark chips; Egg-laying substrate; Moss; Humus; Clay; Soil; Other

- **How often do you clean the enclosure?** Every day; 1 to 6 times a week; Every two weeks or less often; When it's dirty; Other

- **Feeding :(you may select more than one answer):** Commercial feed (pellets); Fruit; Vegetables; Insects; Dried shrimp; Fish; Worms; Other

- **Do you provide vitamin or mineral supplements? Please describe which and how you administer them: (you may select more than one answer)** Vitamin A; Vitamin; Calcium; Other

Part 3 – Owner–Reptile Relationship

Definition
Welfare: A state in which the animal is free from hunger, thirst, discomfort, pain, injury, disease, and fear, and has the freedom to express its normal behavior.

Which definition best describes your reptile? (you may select more than one answer): It is a member of the family; It is a friend; It is a pet; It is a nuisance; None of the above; Other

- **Do you usually talk to your reptile?** No; Yes
- **If yes, how many times a week?** 1; 2; 3 ; 4; 5; >5
- **Do you usually pet your reptile?** No; Yes
- **If yes, how many times a week?** 1; 2; 3 ; 4; 5; >5
- **Do you usually handle/manipulate your reptile? (e.g., placing it on your lap or carrying it):** No; Yes
- **If yes, how many times a week?** 1; 2; 3 ; 4; 5; >5
- **When you approach your reptile, which reactions do you observe most frequently? (you may select more than one answer):** No reaction; It approaches you; It lifts its head; It moves away/tries to escape; It hides; It retracts into its shell; It digs; It vocalizes; It opens its mouth; It bites; Other
- **How would you rate the experience of keeping a reptile as a pet overall?:** Very good; Good; Bad; Very bad; None of the above
- **How often do you take your reptile to the vet?:** Never; Less than once a year; Once a year; Several times a year; Once a month; Several times a month
- **The reptile is seen by a vet for: (you may select more than one answer):** Routine/check-up visits; When I notice a change; Other
- **How would you rate your reptile's welfare? (Welfare: a state in which the animal is free from hunger, thirst, discomfort, pain, injury, disease, and fear, and has the freedom to express its normal behavior) Choose a number from 1 to 5, where 1 is very poor and 5 is very good:** 1; 2; 3; 4; 5

Part 4 – Questions about Behaviour:

Please indicate what you believe are the causes of the following behaviours. For each sentence, select all the options you think are appropriate.

Definitions:

Normal behaviour: a natural behaviour for the species and associated with good welfare.

Welfare: a state in which the animal is free from hunger, thirst, discomfort, pain, injury, disease and fear, and has the freedom to express its natural behaviour.

Behaviour – Possible Cause

- **Do you think that moving around the enclosure exploring objects/people and the environment is a normal behaviour for a reptile?:** Yes; No; Don't know

- **What might it mean if the reptile moves around the enclosure investigating objects/people and exploring the environment? (you may select more than one answer):** Communication; Stress/fear; Escape behaviour;Pain; Dysfunction/disease; Searching for food; Reproductive behaviour; hibernation; Too hot; Too cold; Other

- **Do you think basking in the sunlight, UVB lamp, or heat source with limbs and head extended is a normal behaviour for a reptile?:** Yes; No, Don't Know

- **What might this behaviour mean: "The reptile is basking in the sun, UVB lamp or heat source with limbs and head extended"? (you may select more than one answer):** Communication; Stress/fear; Escape behaviour;Pain; Dysfunction/disease; Searching for food; Reproductive behaviour; hibernation; Too hot; Too cold; Other

- **Do you think persistently trying to push, climb, dig or get around the enclosure's barriers is a normal behaviour for a reptile?:** Yes; No; Don't know

- **What might this behaviour mean: "Frequent attempts to push, climb, dig or get around the enclosure's barriers"? (you may select more than one answer):** Communication; Stress/fear; Escape behaviour;Pain; Dysfunction/disease; Searching for food; Reproductive behaviour; hibernation; Too hot; Too cold; Other

- **Do you think a decrease in activity and/or appetite is a normal behaviour in a reptile?:** Yes; No; Don't Know

- **What might this behaviour mean: "Decrease in activity and/or appetite"? (you may select more than one answer):** Communication; Stress/fear; Escape behaviour;Pain; Dysfunction/disease; Searching for food; Reproductive behaviour; hibernation; Too hot; Too cold; Other

- **Do you think aggression directed at people (e.g., biting or striking) is a normal behaviour in a reptile?** Yes; No; Don't know

- **What might this behaviour mean: "Aggression directed at people, e.g., biting or striking"? (you may select more than one answer):** Communication; Stress/fear; Escape behaviour;Pain; Dysfunction/disease; Searching for food; Reproductive behaviour; hibernation; Too hot; Too cold; Other

- **Do you think that withdrawing head, limbs or tail into the shell in response to human presence or handling (chelonians) is a normal behaviour for a reptile?** Yes; No; Don't know

- **What might this behaviour mean: "Withdrawing the head, limbs or tail into the shell in response to human presence or handling "? (you may select more than one answer):** Communication; Stress/fear; Escape behaviour;Pain; Dysfunction/disease; Searching for food; Reproductive behaviour; hibernation; Too hot; Too cold; Other

- **Do you think open-mouth breathing with the neck extended is a normal behaviour in a reptile?** Yes; No; Don't know

- **What might this behaviour mean: "Open-mouth breathing with neck extended"? (you may select more than one answer):** Communication; Stress/fear; Escape behaviour;Pain; Dysfunction/disease; Searching for food; Reproductive behaviour; hibernation; Too hot; Too cold; Other

- **Do you think that cloacal secretion (urine or faeces) or regurgitation in response to human presence or handling is a normal behaviour in a reptile?** Yes; No; Don't know

- **What might this behaviour mean: "Cloacal secretion (faeces or urine) or regurgitation in response to human presence or handling"? (you may select more than one answer)):** Communication; Stress/fear; Escape behaviour;Pain; Dysfunction/disease; Searching for food; Reproductive behaviour; hibernation; Too hot; Too cold; Other

- **Do you think occupying a dark area, shelter or hide is a normal behaviour for a reptile?** Yes; No; Don't know

- **What might this behaviour mean: "Occupying a dark area, shelter or hide"? (you may select more than one answer)** Communication; Stress/fear; Escape behaviour;Pain; Dysfunction/disease; Searching for food; Reproductive behaviour; hibernation; Too hot; Too cold; Other

Part 5 – Owner Information

- **What type of owner are you?:** Private owner; Breeder; Other

- **Age: __________**

- **Gender:** Female; Male; Prefer not to say

- **Which area of Italy are you from?:** North-West (Liguria, Lombardy, Piedmont, Aosta Valley); North-East (Emilia-Romagna, Friuli Venezia Giulia, Trentino-Alto Adige, Veneto); Central (Lazio, Marche, Tuscany, Umbria); South (Abruzzo, Basilicata, Calabria, Campania, Molise, Apulia);Islands (Sardinia, Sicily)

- **Environment you live in:** City (more than 20,000 inhabitants); Town (between 2,000 and 20,000 inhabitants)**;** Village (fewer than 2,000 inhabitants)**;** Isolated (e.g., countryside house)

- **Level of education:** Primary/middle school; High school; University degree; Postgraduate degree

- **Main occupation:** Work with animals (Veterinarian, Dog trainer, Dog breeder, etc.); Employee; Freelancer; Manual worker; Retired; Student

- **If you are a student, please choose your field of study:** Arts; Scientific disciplines; Humanities; Economics; Other

- **Marital status:** Married; Single (never married); In a domestic partnership; Single; Prefer not to say

- **Type of housing:** Apartment without outdoor space; Apartment with terrace/balcony; House with shared garden; House with private garden

- **How did you find the following survey?:** Trough social pages; It was sent directly by the researchers; My veterinarian suggest me to fill it in; Other

Part 6 – Optional Question

- **Can you explain why you decided to purchase and keep a reptile as a pet?**

- **What are the 3 adjectives that best describe your reptile?**

- **What are the 3 adjectives that best describe your relationship with your reptile?**

- **Do you think your reptile is attached to you?** Very little; Little; Fairly; A lot; Very much

Part 7 – Relationship with Other Pets

- **In this section, please answer the same questions as in the "owner-reptile relationship" section, but referring to one of the other pets you may own.**

- **Do you currently have pets of other species in your home?:** Yes; No

- **Indicate the type of animal(s) you own (you can choose more than one):** Dog; Cat; Rabbit; Budgerigar (Parakeet); Other:

- **Please indicate which animal you will refer to in the following questions (e.g., dog, cat, etc.): ________________**

- **Which definition best describes your pet? (you can choose more than one):** They are a family member; They are a friend; They are a companion animal; They are a nuisance; None of the above; Other:

- **Do you usually talk to your pet?** No; Yes

- **If yes, how many times a week?:** 1; 2; 3; 4; 5; >5

- **Do you usually pet your pet? :** No; Yes

- **If yes, how many times a week?** 1; 2; 3; 4; 5; >5

- **Do you usually handle/manipulate your pet? (e.g., holding it in your lap or carrying it)** No; Yes

- **If yes, how many times a week?** 1; 2; 3; 4; 5; >5

- **What are the 3 adjectives that best describe your pet?**

- **What are the 3 adjectives that best describe your relationship with your pet?**

- **Do you think your pet is attached to you?:** Very little; Little; Fairly; A lot; Very much

Table S1. Full demographic information of participants (N=91)

Variable	Absolute frequency	Relative frequency (%)
**Gender**		
Female	67	73.6
Male	22	24.2
Prefer not to say	2	2.2
**Region**		
Center	39	42.9
North-Est	14	15.4
North-West	28	30.8
South	5	5.5
Islands	4	4.4
**Urbanization**		
City	38	41.8
Town	35	38.5
Isolated	11	12.1
Village	6	6.6
Prefer not to say	1	1.1
**Education**		
University degree	34	37.4
Post university	21	23.1
Middle school	5	5.5
High-school	29	31.9
Prefer not to say	2	2.2
**Occupation**		
Employee	34	37.4
Works with animals	18	19.8
Self-employed	18	19.8
Unemployed, student or pensioner	9	9.9
Laborer	8	8.8
Prefer not to say	4	4.4
**Marital Status**		
Married	45	49.5
Cohabitating	32	35.2
Single	8	8.8
Prefer not to say	6	6.6
**House**		
With private garden	80	87.9
With terrace/balcony	10	10.9
With shared garden	9	9.8
Prefer not to say	1	1.1

Table S2. Stepwise Model Selection Process (AIC-Based) for Predictors of Bond Type. This table presents the AIC-based stepwise selection process used to identify the most important predictors of bond type in the logistic regression model. The stepwise procedure systematically removed non-significant predictors one by one until the final model was reached.

Step	Removed Variable	Degrees of Freedom (Df)	Deviance	AIC After Removal
1	**Profession**	3	114.72	150.72
2	**Region**	3	114.78	150.78
3	**Acquisition**	2	114.52	152.51
4	**Civil Status**	2	114.60	152.60
5	**Species**	2	114.71	152.71
6	**Education**	2	114.77	152.77
7	**Urbanization**	2	115.33	153.33
8	**Gender**	1	114.46	154.46
9	**Age**	1	114.46	154.46
10	**House Type**	1	115.82	155.82
**Final Model**	**Only "Environment" remains**	—	**118.40**	**122.40**
